# The Role of 3D CT Imaging in the Accurate Diagnosis of Lung Function in Coronavirus Patients

**DOI:** 10.3390/diagnostics12030696

**Published:** 2022-03-12

**Authors:** Ibrahim Shawky Farahat, Ahmed Sharafeldeen, Mohamed Elsharkawy, Ahmed Soliman, Ali Mahmoud, Mohammed Ghazal, Fatma Taher, Maha Bilal, Ahmed Abdel Khalek Abdel Razek, Waleed Aladrousy, Samir Elmougy, Ahmed Elsaid Tolba, Moumen El-Melegy, Ayman El-Baz

**Affiliations:** 1 BioImaging Laboratory, Department of Bioengineering, University of Louisville, Louisville, KY 40292, USA; ishawky@fci.luxor.edu.eg (I.S.F.); a.sharafeldeen@louisville.edu (A.S.); mohamed.elsharkawy@louisville.edu (M.E.); ahmed.soliman@louisville.edu (A.S.); ahmahm01@louisville.edu (A.M.); 2 Electrical, Computer, and Biomedical Engineering Department, College of Engineering, Abu Dhabi University, Abu Dhabi 59911, United Arab Emirates; mohammed.ghazal@adu.ac.ae; 3 College of Technological Innovation, Zayed University, Dubai 19282, United Arab Emirates; fatma.taher@zu.ac.ae; 4 Department of Diagnostic Radiology, Faculty of Medicine, Mansoura University, Mansoura 35516, Egypt; mahabilal@mans.edu.eg (M.B.); arazek@mans.edu.eg (A.A.K.A.R.); 5 Computer Science Department, Faculty of Computers and Information, Mansoura University, Mansoura 35516, Egypt; waleed_m_m@mans.edu.eg (W.A.); mougy@mans.edu.eg (S.E.); ast@astolba.com (A.E.T.); 6 The Higher Institute of Engineering and Automotive Technology and Energy, New Heliopolis 11829, Cairo, Egypt; 7 Department of Electrical Engineering, Assiut University, Assiut 71511, Egypt; moumen@aun.edu.eg

**Keywords:** COVID-19, SARS-CoV-2, machine learning, neural network, Computer Assisted Diagnosis (CAD), Markov–Gibbs Random Field (MGRF)

## Abstract

Early grading of coronavirus disease 2019 (COVID-19), as well as ventilator support machines, are prime ways to help the world fight this virus and reduce the mortality rate. To reduce the burden on physicians, we developed an automatic Computer-Aided Diagnostic (CAD) system to grade COVID-19 from Computed Tomography (CT) images. This system segments the lung region from chest CT scans using an unsupervised approach based on an appearance model, followed by 3D rotation invariant Markov–Gibbs Random Field (MGRF)-based morphological constraints. This system analyzes the segmented lung and generates precise, analytical imaging markers by estimating the MGRF-based analytical potentials. Three Gibbs energy markers were extracted from each CT scan by tuning the MGRF parameters on each lesion separately. The latter were healthy/mild, moderate, and severe lesions. To represent these markers more reliably, a Cumulative Distribution Function (CDF) was generated, then statistical markers were extracted from it, namely, 10th through 90th CDF percentiles with 10% increments. Subsequently, the three extracted markers were combined together and fed into a backpropagation neural network to make the diagnosis. The developed system was assessed on 76 COVID-19-infected patients using two metrics, namely, accuracy and Kappa. In this paper, the proposed system was trained and tested by three approaches. In the first approach, the MGRF model was trained and tested on the lungs. This approach achieved 95.83% accuracy and 93.39% kappa. In the second approach, we trained the MGRF model on the lesions and tested it on the lungs. This approach achieved 91.67% accuracy and 86.67% kappa. Finally, we trained and tested the MGRF model on lesions. It achieved 100% accuracy and 100% kappa. The results reported in this paper show the ability of the developed system to accurately grade COVID-19 lesions compared to other machine learning classifiers, such as k-Nearest Neighbor (KNN), decision tree, naïve Bayes, and random forest.

## 1. Introduction

**Figure 1:**
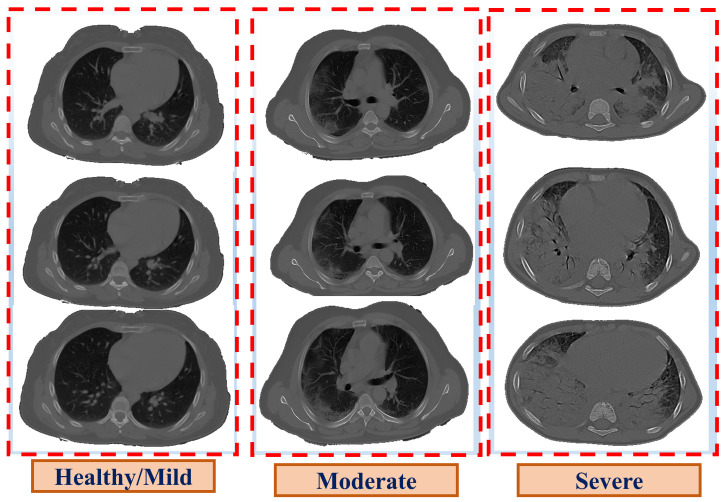
Illustrative examples of the three grades of COVID-19.

At the end of 2019, a new infectious disease, named Coronavirus Disease 2019 (COVID-19), emerged in Wuhan, China, and spread throughout the world [1]. The incubation period for COVID-19 (i.e., the time between exposure and the symptoms) is two to fourteen days, and symptoms often appear on the fourth or fifth day [2]. The symptoms of COVID-19 differ from one person to another based on the severity level of the disease. Many people infected with COVID-19 have mild to moderate symptoms and recover without the need for hospitalization, unlike severe symptoms that require mechanical ventilation, which may lead to death. However, it is possible to have a COVID-19 infection without showing any symptoms [3]. According to the Centers for Disease Control and Prevention (CDC), 40% of COVID-19 cases are asymptomatic [4] while COVID-19 prevalence [5] was estimated at 115.6 million with a 2.22% mortality rate. COVID-19’s symptoms are classified into three categories [6]: mild, moderate, and severe; their symptoms are discussed in [7]. Severe disease is characterized by severe pneumonia, causing significant respiratory compromise and hypoxemia, requiring supplemental oxygen, and hospitalization, and can progress to pulmonary fibrosis (lung scarring), shock, and death [7]. Therefore, early diagnosis and grading of COVID-19 infection is vital to prevent any health complications and, thus, reduce the mortality rate. Several radiological modalities, such as Computed Tomography (CT), which is the most effective tool used to detect lung anomalies, particularly in its early stages [8,9], are employed as assistive toold in diagnosing the severity of COVID-19, ranging from the plain chest or the patchy involvement of one or both lungs in the mild or moderate cases, to a pulmonary infiltrate, called white lung, in extreme cases [10], as presented in Figure 1.

For mild to moderate cases, medical attention or non-invasive ventilation is utilized as a treatment method while mechanical ventilation is adopted in severe cases to help the patients breathe, due to Acute Respiratory Distress Syndrome (ARDS). Although CT has some limitations, such as poor specificity and difficulty differentiating between anomalies and pneumonia during influenza or adenovirus infections [11], its high sensitivity makes it an excellent tool for determining the disease in patients with confirmed COVID-19.

In recent years, multiple Computer-Aided Diagnosis (CAD) systems employed machine learning approaches to classify COVID-19 as well as grade the severity of COVID-19. For example, Barstugan et al. [12] proposed a system to diagnose COVID-19 in CT images against normal ones. First, predefined sets of features were extracted from different patch sizes. This set included the Gray-Level Co-occurrence Matrix (GLCM), Gray-Level Run Length Matrix (GLRLM), Local Directional Pattern (LDP), Discrete Wavelet Transform (DWT), and Gray-Level Size Zone Matrix (GLSZM). Subsequently, these features were classified using a Support Vector Machine (SVM) classifier. Ardakani et al. [13] presented a CAD system to diagnose COVID-19 and non-COVID-19 pneumonia using 20 radiomic features that extracted from CT images. These features were used to capture the distributions, locations, and patterns of lesions, and subsequently were fed to five classifiers, namely, SVM, K-Nearest Neighbor (KNN), decision tree, naïve Bayes, and ensemble. Their results showed that the ensemble classifier gave the best accuracy against the other classifiers. On the other hand, some literature used Deep Learning (DL) techniques to enhance the accuracy of the COVID-19 classification. For example, Zhang et al. [14] used an AI method to detect infected lung lobes for diagnosing the severity of COVID-19 pneumonia on chest CT images. They used a pretrained DL model to extract the feature maps from the chest CT images and provide the final classification. Their test was based on the the number of infected lobes of the lungs, and their severity grading values. Qianqian et al. [15] used deep neural networks to detect anomalies in the chest CT scans of COVID-19 patients by extracting CT features and estimating anomalies of pulmonary involvement. Their system consisted of three processes to diagnose the COVID-19 patients, namely, the detection, segmentation, and localization information of lesions in the CT lung region. They utilized 3D U-Net [16] and MVP-Net networks [17]. Goncharov et al. [18] used a Convolution Neural Network (CNN) to identify COVID-19-infected individuals using CT scans. First, they identified the COVID-19-infected lung, then they made severity quantifications to study the state of patients and provide them with suitable medical care. Kayhan et al. [19] identified the stages of infection for COVID-19 patients with pneumonia: mild, progressive, and severe using CT images. They used a modified CNN and KNN to extract the features from the lung inflammation and then make a classification. The system identified the severity of COVID-19 in two steps. In the first step, the system calculated lesion severity in CT images of a confirmed COVID-19 patient. In the second step, the system classified the pneumonia level of a confirmed COVID-19 patient using the modified CNN and KNN. Shakarami [20] diagnosed COVID-19 using CT and X-ray images. First they used a pretrained AlexNet [21] to extract the features from CT images. Secondly, a Content-Based Imaged Retrieval system (CBIR) was used to find the most similar cases to carry out more statistical analyses on patient profiles that were similar. Lastly, they applied a majority voting technique on the outputs of CBIR for final diagnosing. Another study by Zheng et al. [22] proposed a CAD system based on a 3D CNN using CT images. This system segmented the lung based on a 2D U-Net. Subsequently, a CT image and its mask were fed to a 3D CNN, which consisted of three stages. First, a 3D convolution layer with a 5×5×7 kernel size was used. Subsequently, two 3D residual blocks were adopted. Finally, a prediction classifier with a softmax activation function in its fully connected layer was employed to detect COVID-19 in the CT scan. The report accuracy of the system was 90.1%. Wang et al. [23] presented a CAD system to segment and classify COVID-19 lesion in CT images. The latter utilized well know CNNs for segmentation, namely U-Net, 3D U-Net++ [24], V-Net [25], and Fully Convolutional Networks (FNC-8s) [26]; as well as classification; namely, inception [27], Dual Path Network (DPN-92) [28], ResNet-50, and attention ResNet-50 [29]. Their results showed that the best model for lesion segmentation and classification was 3D U-Net++ and ResNet-50, respectively.

Most of the works suffer from some drawbacks: (1) the existing work used the deep learning techniques, which depends on convolution layers to extract the feature maps, which may not be related to COVID-19 patients. (2) Most CAD systems tended to offer cruder outputs, such as the existence of COVID-19 or not. Therefore, this paper focuses on the development of a CAD system using CT images to help physicians accurately grade COVID-19 infections, allowing them to prioritize patient needs and initiate appropriate management. This tool will guarantee the safety of patients by directing them to the right way and prioritize the usage of medical resources. Our system grades COVID-19 into one of the three categories: healthy/mild, moderate, and severe. First, the lungs are segmented from CT scans based on an unsupervised technique that adapts the first order appearance model in addition to morphological constraints based on a 3D rotation invariant Markov–Gibbs Random Field (MGRF). Then, the tissues of the segmented lungs are modeled using the 3D rotation invariant MGRF model to extract three distinguishable features. These include the Gibbs energy, estimated based on tuning the model parameters for each grade separately. Subsequently, a Cumulative Distribution Function (CDF) is created and sufficient statistical features are extracted. Namely, the 10th through 90th CDF percentiles with 10% increments. Finally, a Neural Network (NN) is employed and fed with the concatenation of these features to make the final diagnosis. In addition, we applied three approaches to tune MGRF parameters. In the first approach, the system was trained and tested on the lung. In the second approach, the system was trained and tested on lesions. In the third approach, the system was trained on lesions and tested on lungs.

## 2. Methods

**Figure 2:**
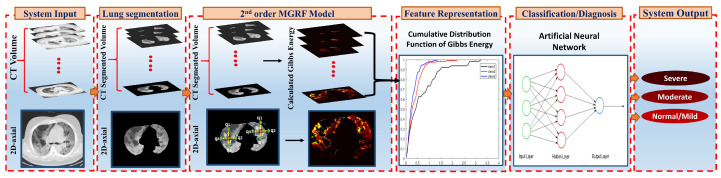
An illustrative framework for the proposed CAD system to detect the severity of COVID-19 through the CT images.

To achieve the main goal of this project, we proposed the CAD system that is shown in Figure 2. This CAD system consists of three major steps: (i) extracting the lung region from 3D CT images; (ii) developing a rotation, translation, and scaling invariant MGRF model to learn the appearance model of the infected lung region for a different level of severity (mild, moderate, and severe); and (iii) developing a Neural Network (NN)-based fusion and diagnostic system to determine whether the grade of lung infection is mild, moderate, or severe.

### 2.1. Lung Segmentation

**Figure 3:**
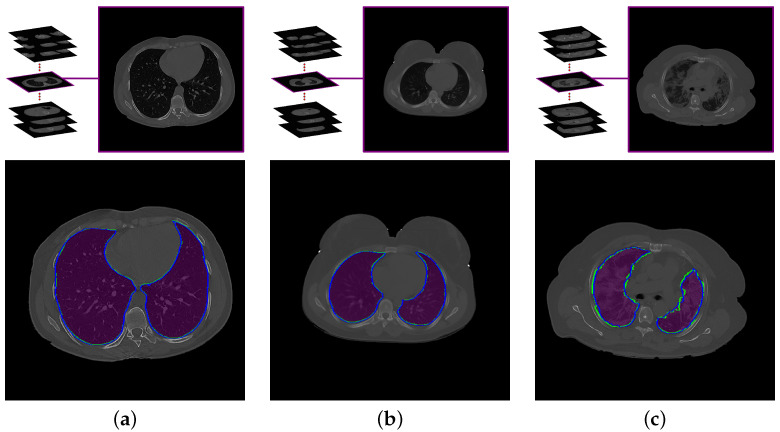
An illustrative example of the proposed segmentation approach for (**a**) healthy/mild, (**b**) moderate, and (**c**) severe COVID-19 infections. Note that the blue (green) border represents our segmentation (ground truth).

To obtain the most accurate labeling of the effected lung, we must first limit the region of interest to the lungs, properly, excluding non-lung tissue that could otherwise be misidentified as pathological. Thus, the first step in the proposed system is to delineate the boundaries of the three-dimensional lung region in the CT images, as near as possible to how a professional radiologist would perform this task. Some lung tissues, such as arteries, veins, and bronchi, have radiodensity similar to tissues elsewhere in the chest. Therefore, segmentation must consider not only the image gray level, but also the spatial relationship of the CT signal and image segments in 3D, so that the details of the lungs are preserved. To achieve this step, we used our lung segmentation approach previously published in [30], which incorporates both the radiodensity distribution of lung tissues and the spatial interaction among neighboring voxels within the lung. Figure 3 demonstrates the segmentation results of this approach for three subjects with different grades of lung infection (mathematical details of this approach are presented in [30]).

### 2.2. MGRF-Based Severity Detection Model

In order to capture the inhomogeneity that may be caused by COVID-19 infection, a Markov–Gibbs Random Field (MGRF) model [31,32,33] is utilized, which is one of the mathematical models that shows a high ability to capture the inhomogeneity in the virtual appearance model. An instance of an MGRF is specified by an interaction graph, defining which voxels are considered neighbors, and a Gibbs Probability Distribution (GPD) on that graph, which gives the joint probability density of gray levels in a voxel neighborhood. Under a weak condition of strictly positive probabilities of all the samples, the full GPD may be factored into subcomponents corresponding to the cliques, or complete subgraphs, of the interaction graph [31]. Historically, applications of MGRF to image processing have worked to improve their ability to express the richness of the visual appearance by careful specification of the GPD, and to develop powerful algorithms for statistical inference.

This paper introduces a class of an MGRF model that is invariant under translation and contrast stretching [31]. It is a generalization of the classical Potts model onto multiple third-order interactions. Learning of model parameters is conducted by adapting a fast analytical framework originally devised for generic second-order MGRF [31]. The proposed higher-order models allow for fast learning of most patterns that are characteristic of the visual appearance of medical images. The proposed nested MGRF models and its learning are introduced as follows.

Let G be the set of grayscale images on a pixel raster R={0,…,X−1}×{0,…,Y−1}, i.e., the set of mappings from R to discrete gray values Q={0,…,Q−1}. For any MGRF model, there is a corresponding probability that g∈G is generated by that model, namely the Gibbs probability distribution P(g), where (for the normalized GPD) $\sum_{g\in G}P\left(g\right)=1$. In practice P(g) is factored over the maximal cliques of an interaction graph on the pixel raster. The GPD is then completely specified by the set of cliques and their corresponding Gibbs potentials (logarithmic factors).

A translation invariance, *K*-order interaction structure on R, is a system of clique families, $C_{a},\;a=1,\ldots ,A$. Each family comprises cliques of one particular shape, and the clique origin nodes include every pixel in R. The corresponding *K*-variate potential function, $V_{a}\left(g\left(r^{\prime }\right):\;r^{\prime }\in c_{a:r}\right)$, depends on ternary ordinal relationships between pixels within the clique. The GPD of the translation- and contrast-invariant MGRF then factors as:

(1) $$P\left(g\right)=\frac{1}{Z}P_{0}\exp\left(-\sum_{a=1}^{A}\sum_{c_{a:r}\in C_{a}}V_{a}\left(g\left(r^{\prime }\right):\;r^{\prime }\in c_{a:r}\right)\right).$$

The inner sum $\sum_{c_{a:r}\in C_{a}}V_{a}\left(g\left(r^{\prime }\right):\;r^{\prime }\in c_{a:r}\right)$ is called the Gibbs energy and denoted E(g). The partition function $Z=\sum_{g\in G}\exp\left(-E\left(g\right)\right)$ normalizes the GPD over the G. $P_{0}$ denotes the base probability model. Given a training image $g^{\circ }$, the Gibbs potentials for the generic low- and high-order MGRF models are approximated in the same way as for the generic second-order MGRF accounting for signal co-occurrences in [31]:

$$V_{a}=-\lambda_{a}\left(F_{a}\left(g^{\circ }\right)-F_{a:\mathrm{ref}}\right).$$

Here, $F_{a}\left(g^{\circ }\right)$ is the normalised histogram of gray value tuples over $c_{a}$ for the image $g^{\circ }$, while $F_{a:\mathrm{ref}}$ denotes the normalised histogram component for the base random field. In principle, the values $F_{a:\mathrm{ref}}$ can be computed from the marginal signal probabilities or easily evaluated from generated samples of this base probability distribution. The scaling factor $\lambda_{a}$ is also computed analytically [31].

To model lung appearance, a signal co-occurrence-based, multiple pair-wise MGRF model is first employed to learn both the shapes of the cliques and potentials from a set of training lung images. Learning the clique families follows [31] by analyzing the family-wise partial Gibbs energies over a large search pool of potential clique families. The least energetic cliques, which best capture the pixel interactions of the training image set, were selected by unimodal thresholding of the empirical distribution of the family-wise interaction energies [31]. The selection threshold corresponds to the distribution curve to the point at the maximal distance from a straight line from the peak energy to the last non-empty bin of the energy histogram.

**Figure 4:**
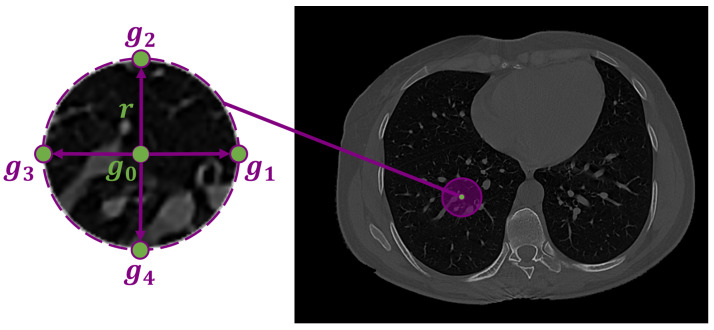
Fourth-order LBP structure, $g_{0}$ is the central pixel, $g_{1}$, $g_{2}$, $g_{3}$, and $g_{4}$ are the four neighbours, and *r* is the radius.

The infected region in the lung tissues is represented by the pixel-wise Gibbs energy of the proposed high-order MGRF. This Gibbs energy is computed by summing the potentials across all characteristic cliques for each pixel in the test subject. The proposed high-order MGRF model is based on using a heuristic fixed neighborhood structure (circular shape) to model the COVID-19 lung lesions. Figure 4 shows the high-order neighborhood structure with signal configurations: $\left\{B\left(g_{0}-g_{1}\right),B\left(g_{0}-g_{2}\right),B\left(g_{0}-g_{3}\right),B\left(g_{0}-g_{4}\right),N\left[g_{0},g_{1},g_{2},g_{3},g_{4}\right]\right\}$. *B* denotes the binary ordinal interactions,

(2) $$B\left(g_{0}-g_{1}\right)=1\;\mathrm{if}\;\left|g_{0}-g_{1}\right|>1$$

*N* denotes the number of signals greater than *T*; there are six possible values, from 0 to 5 (to discriminate between the lung/non-lung LBPs). In total, $2^{4}\times 6$ signal configurations. The threshold *T* is learned from the training image.

Algorithm 1 presents the details of learning LBPs. The energy for each pixel is the sum of potentials over 5 cliques (LBP circular structure) involved with this pixel, and then get the normalized energy.
**Algorithm 1:** Learning the 4th-order LBPs.(1)For the training COVID-19 CT image, compute the vector of empirical probabilities (frequencies), $F_{r}=\left[f_{r}\left(h\right):h=1,\ldots ,96\right]$ of the above signal interactions.(2)Compute the frequency $F_{ref:r}=\left[f_{ref:r}\left(h\right):h=1,\ldots ,96\right]$ of the same signal interactions from the synthesized image, sampled from the learned second-order MGRF, acting as a base field.(3)Compute the potentials, $V_{r}\left(h\right)=\lambda_{h}$×$\left(f_{r}\left(h\right)-f_{core:r}\left(h\right)\right)$, $\lambda_{h}=1/(f_{core:r}\left(h\right)$× $\left(1-f_{core:r}\left(h\right))\right)$.(4)Compute total Gibbs energy of image for candidate radius r=1:1:10, choose *r* with the largest Gibbs energy.

### 2.3. Feature Representation and Classification System

**Figure 5:**
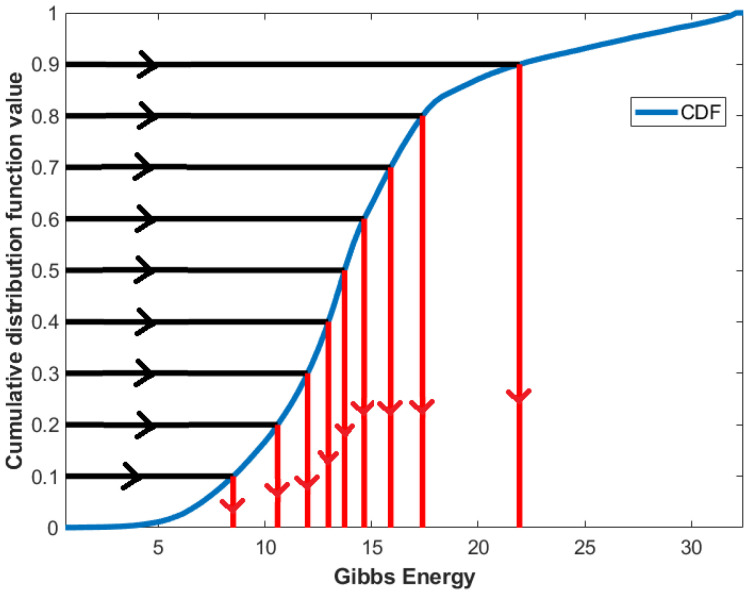
An illustrative example of the estimation of CDF percentile feature from CDF.

For a better representation of Gibbs energy, statistical features are employed, namely, the 10th–90th percentiles with 10% increments. These features are extracted by first calculating the CDF, then interpolating the feature values at 0.1–0.9, as presented in Figure 5.

Then, an NN-based system is built and fed with the concatenation of the CDF percentiles, extracted from the diagnostic findings of the three Gibbs energies, estimated from the three MGRF-based trained models at each grade separately, as shown in Figure 2. This network is trained based on the Levenberg–Marquardt optimization algorithm [34], which considers the fastest backpropagation algorithm. Algorithm 2 presents the basic steps of NN training. This network is tuned by running multiple experiments to select the best NN hyperparameters. These include the number of hidden layers and the number of neurons in each hidden layer. The setup of this network involves three hidden layers with 27, 21, and 11 neurons in each layer, respectively (searching from 2 to 100 neurons).
**Algorithm 2:** Backpropagation algorithm.(1)The value of weights in all layers are initialized randomly.(2)The values of each neuron in the hidden layer and output layer are calculated.(3)The weights in a neural network are updated using Levenberg–Marquardt optimization.(4)Step 2 is repeated until one of the following conditions is achieved:
Reaching the maximum number of epochs.Exceeding the maximum specified time.Achieving the target performance.

## 3. Experimental Results

### 3.1. Patient Data

We tested our CAD system using COVID-19 positive CT scans collected from Mansoura University, Egypt. This database contains CT images of 76 patients divided into three categories healthy/mild, moderate, and severe.

The research study followed all of the required procedures and were performed in accordance with the relevant guidelines where the Institutional Review Board (IRB) at Mansoura University approved the study and all methods. Informed consent from the patients were obtained, and for the patients who passed away, informed consent was obtained from the legal guardian/next of kin of the deceased patient. The dataset contains 15 healthy/mild cases, 35 moderate cases, and 26 severe cases.

### 3.2. Evaluation Metrics

We used three evaluation metrics—precision, recall, and *F*1-score—for each individual class. For each class *i*, we calculated the true positive ($TP_{i}$), false positive ($FP_{i}$), true negative ($TN_{i}$), and false negative ($FN_{i}$). Then, we calculated the three evaluation matrices for each class as follows:

(3) $$Precision_{i}=\frac{TP_{i}}{TP_{i}+FP_{i}}$$

(4) $$Recall_{i}=\frac{TP_{i}}{TP_{i}+FN_{i}}$$

(5) $$F1-score_{i}=\frac{2TP_{i}}{2TP_{i}+FP_{i}+FN_{i}}$$

Moreover, we calculated the overall accuracy and Cohen kappa for all classes as follows:

(6) $$Overall-accuracy=\frac{\sum_{c=1}^{k}TP_{i}}{N}$$

(7) $$Kappa=\frac{P_{o}-P_{e}}{1-P_{e}}$$

where *k* is the number of classes, *N* is the total number of test data, $P_{o}$ denotes the observed relative agreement between raters, and $P_{e}$ denotes the theoretical probability of random agreement.

### 3.3. The Performance of the Proposed System

We conducted our proposed system using three different methodologies. The first method (lung model) estimates Gibbs energy by training and testing the model on the patient’s lung. The second method (hybrid model) calculates Gibbs energy by training the model on the patient lesion. Then the model is tested on the lung. The third method (lesion model) estimates Gibbs energy by training and testing the model on the lesion. The evaluation of these models is demonstrated in Table 1. As shown in the table, our proposed lesion model performance outperforms the other two models (i.e., lung and hybrid models) with an overall accuracy and a kappa of 100% and 100%, respectively. Thus, the reported results show that the lesion model is the best model for the proposed system. Moreover, to highlight the promise of the proposed NN-based system, different statistical machine learning classifiers were employed in the lung, lesion, and hybrid models separately. For example, a KNN classifier was utilized, which achieved an overall accuracy of 79.17%, 79.17%, and 66.67%, respectively, while the Kappa statistics were 66.1%, 67.48%, and 49.2%, respectively. In addition, SVM classifier achieved overall accuracies of 70.83%, 79.17%, and 70.83%, respectively, while the Kappa statistics were 56.92%, 66.94%, and 55.56%, respectively. A naïve Bayes classifier was also employed, which achieved overall accuracies of 54.17%, 91.67%, and 58.33%, respectively, while the Kappa statistics were 34.32%, 87.1%, and 39.39%, respectively. The decision tree classifier was adapted as well and achieved overall accuracies of 66.67%, 79.17%, and 62.5%, respectively; and Kappa statistics of 48.8%, 66.1%, and 36.84%, respectively. Finally, a random forest classifier was used and achieved overall accuracies of 83.33%, 87.5%, and 75%, respectively; and Kappa statistics of 73.98%, 79.83%, and 58.62%, respectively. From these results, we can conclude that our proposed NN-based system achieves high accuracy when compared to other classifiers.

**Figure 6:**
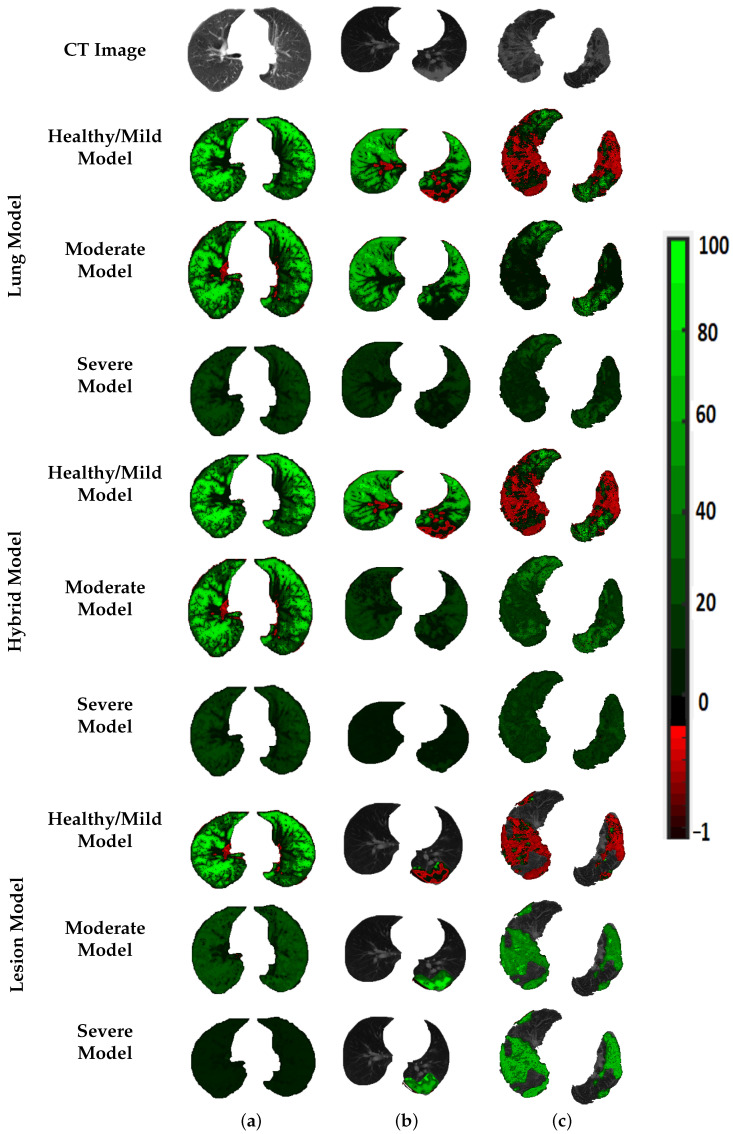
An illustrative color map example of Gibbs energies for (**a**) healthy/mild, (**b**) moderate, and (**c**) severe; tuned using healthy/mild, moderate, or severe COVID-19 lesions; applied to the lung (2nd–4th rows), hybrid (5th–7th rows), and lesion (8th–10th rows) approaches.

**Figure 7:**
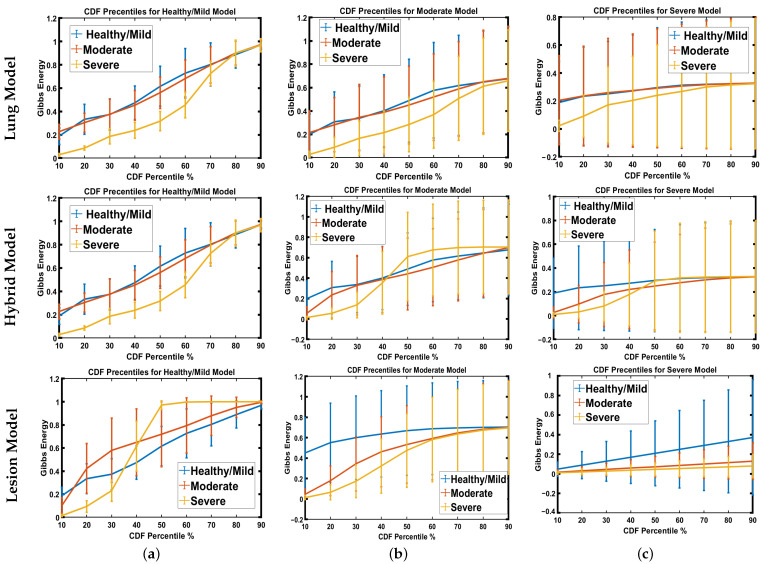
Estimated error average of CDF percentiles for three grades when tuning MGRF parameters using (**a**) healthy/mild, (**b**) moderate, or (**c**) severe lesion infection, applied to lung (upper row), hybrid (middle row), and lesion (lower row) approaches.

To prove that the results shown in the previous tables are not coincidental, the estimated Gibbs energy is represented by a color map, see Figure 6. As demonstrated in the figure, Gibbs energy for each grade is higher than the other two grades when the model is tuned using the same grade. For example, Gibbs energy for the healthy/mild case is higher than that of the moderate and severe cases when tuned using healthy/mild cases, and applied to the lesion model. The same goes for moderate and severe MGRF tuning. This shows the reported results in recognition of three grades, especially when applied to the lesion model. Since there are variable resolutions of the CT images in the dataset, we employed CDF percentiles as novel scale-invariant representations of the estimated Gibbs energy, acceptable for all data collection techniques. Figure 7 shows the average error of the CDF percentiles for three grades when tuning MGRF parameters using healthy/mild and moderate and severe lesions, applied to the three models: lung, hybrid, and lesion models. As shown in the figure, the CDF percentiles of the proposed system, when applied to the lesion model, are more separable than the other two models, demonstrating the efficiency of the lesion model compared to lung and hybrid models. This establishes the attainable accuracy of the proposed lesion model.

## 4. Discussion

Patients with severe COVID-19 suffer from significant respiratory compromises and even ARDS. A substantial fraction of COVID-19 inpatients develop ARDS, of whom, 61% to 81% require intensive care [40]. COVID-19 can also induce a systemic hyperinflammatory state, leading to multiorgan dysfunction, such as heart failure and acute renal failure. Consequently, COVID-19 patients admitted to the Intensive Care Unit (ICU) requiring mechanical ventilation have alarmingly high mortality rates. Early in the pandemic, the mortality rate reached 97% [41,42]. Therefore, it is vital to identify patients with severe COVID-19 lung pathology before they progress to ARDS, respiratory failure, or systemic hyperinflammation, all of which greatly increase the risk of death. Medical resources in health systems across the world have been severely strained. Fast, automated, and accurate assessments of lung CT scans can aid medical care by reducing the burden on medical staff to interpret images, providing rapid interpretations, and making scan interpretations more objective and reliable. In this study, we showed that our system can successfully classify patients into either normal-to-mild, moderate, or severe cases, with accuracies of 92–100% depending on which of our three testing and training approaches is used. Our lesion model produced perfect accuracy in this dataset. This compares very favorably to existing AI systems for analyzing chest imaging in COVID-19 patients. A number of previous studies have also applied AI to chest X-rays or CT scans [13,14,15,16,17,18,19,20,21,22,23,24,25,26,27,28,29,43,44,45,46,47,48,49,50,51,52,53]. These studies achieve accuracies between 90.1% and 95%. Various machine learning techniques were employed, such as convoluted neural networks and deep learning approaches. Some have also used fused imaging data with clinical, demographic, and laboratory data to enhance their systems [44]. While this can improve the accuracy of such systems, most of them suffer from the same drawbacks: (1) the existing work uses deep learning techniques which depend on convolution layers to extract feature maps that may not be related to the pulmonary pathophysiology of COVID-19 patients. (2) Most CAD systems tended to offer cruder outputs, such as the presence of COVID-19 or not. Since its debut, AI has proved to be beneficial in medical applications and has been generally accepted because of its great predictability and precision. Clinical results can be improved by using AI in conjunction with thoracic imaging and other clinical data (PCR, clinical symptoms, and laboratory indicators) [54]. During the COVID-19 diagnostic stage, AI may be utilized to identify lung inflammation in CT medical imaging. AI also can be used to segment regions of interest from CT images. Therefore self-learned features can be easily retrieved for diagnosis or for any other use. Through the fusion of imaging data inside an AI framework, multimodal data, whether clinical or epidemiological data, may be constructed to detect and treat COVID-19 patients, in addition to potentially stopping this pandemic from spreading.

## 5. Conclusions

In conclusion, our results demonstrate that AI can be used to grade the severity level of COVID-19 by analyzing the chest CT images of COVID patients. As we have shown, the high mortality rate is related to pneumonia severity on chest CT images. Therefore, our CAD system will be utilized to detect the severity of COVID-19. Then, the patient will be directed to the correct treatment. This will lead to a reduction in the mortality rate of COVID-19. In the future, we plan to collect more data and validate our developed system on separate data, as well as include demographic markers in our analysis.

## Figures and Tables

**Table 1:** Comparison between the proposed system and different machine learning classifiers using lung, hybrid, and lesion models.

			Class Evaluation			Overall Evaluation	
	Classifier	Class	Recall	Precision	F1-Score	Overall Accuracy	Kappa
**Lung Model**	Random Forest [35]	Healthy/Mild	80%	66.67%	72.73%	83.33%	73.98%
		Moderate	81.82%	81.82%	81.82%		
		Severe	87.50%	100%	93.33%		
	Decision Trees [36]	Healthy/Mild	80%	57.14%	66.67%	66.67%	48.8%
		Moderate	63.64%	70%	66.67%		
		Severe	62.50%	71.43%	66.67%		
	Naive Bayes [37]	Healthy/Mild	100%	41.67%	58.82%	54.17%	34.32%
		Moderate	9.09%	50%	15.38%		
		Severe	87.50%	70%	77.78%		
	SVM [38]	Healthy/Mild	100%	62.50%	76.92%	70.83%	56.92%
		Moderate	45.45%	83.33%	58.82%		
		Severe	87.5%	70%	77.78%		
	KNN [39]	Healthy/Mild	60%	75%	66.67%	79.17%	66.1%
		Moderate	90.91%	71.43%	80%		
		Severe	75%	100%	85.71%		
	Proposed System	Healthy/Mild	100%	100%	**100%**	95.83 **%**	93.39 **%**
		Moderate	100%	91.67%	95.65%		
		Severe	87.50%	100%	93.33%		
**Hybrid Model**	Random Forest [35]	Healthy/Mild	40%	66.67%	50%	75%	58.62%
		Moderate	90.91%	66.67%	76.92%		
		Severe	75%	100%	85.71%		
	Decision Trees [36]	Healthy/Mild	20%	50%	28.57%	62.50%	36.84%
		Moderate	81.82%	56.25%	66.67%		
		Severe	62.50%	83.33%	71.43%		
	Naive Bayes [37]	Healthy/Mild	80%	44.44%	57.14%	58.33%	39.39%
		Moderate	27.27%	60%	37.50%		
		Severe	87.50%	70%	77.78%		
	SVM [38]	Healthy/Mild	80%	80%	80%	70.83%	55.56%
		Moderate	54.55%	85.71%	66.67%		
		Severe	87.5%	58.33%	70%		
	KNN [39]	Healthy/Mild	60%	42.86%	50%	66.67%	49.2%
		Moderate	54.55%	66.67%	60%		
		Severe	87.5%	87.5%	87.50%		
	Proposed System	Healthy/Mild	100%	100%	100%	91.67 **%**	86.67 **%**
		Moderate	100%	84.62%	91.67%		
		Severe	75%	100%	85.71%		
**Lesion Model**	Random Forest [35]	Healthy/Mild	100%	100%	100%	87.5%	79.83%
		Moderate	100%	78.57%	88%		
		Severe	62.5%	100%	76.92%		
	Decision Trees [36]	Healthy/Mild	80%	100%	88.89%	79.17%	66.1%
		Moderate	90.91%	71.43%	80%		
		Severe	62.5%	83.33%	71.43%		
	Naive Bayes [37]	Healthy/Mild	100%	100%	100%	91.67%	87.1%
		Moderate	81.82%	100%	90%		
		Severe	100%	80%	88.89%		
	SVM [38]	Healthy/Mild	80%	100%	88.89%	79.17%	66.94%
		Moderate	81.82%	75%	78.26%		
		Severe	75%	75%	75%		
	KNN [39]	Healthy/Mild	100%	100%	100%	79.17%	67.48%
		Moderate	72.73%	80%	76.19%		
		Severe	75%	66.67%	70.59%		
	Proposed System	Healthy/Mild	100%	100%	100%	100 **%**	100 **%**
		Moderate	100%	100%	100%		
		Severe	100%	100%	100%		

## Data Availability

Data are made available through the corresponding author upon reasonable request.
